# GILZ as a Mediator of the Anti-Inflammatory Effects of Glucocorticoids

**DOI:** 10.3389/fendo.2015.00170

**Published:** 2015-11-09

**Authors:** Simona Ronchetti, Graziella Migliorati, Carlo Riccardi

**Affiliations:** ^1^Section of Pharmacology, Department of Medicine, University of Perugia, Perugia, Italy

**Keywords:** glucocorticoids, GILZ, inflammation, immune cells, transcription factor

## Abstract

Glucocorticoid-induced leucine zipper (GILZ) is a dexamethasone-inducible gene that mediates glucocorticoid (GC) actions in a variety of cell types, including many cells of immune system. In particular, GILZ can control T cell activities, such as activation and differentiation, mainly through its ability to homo- and hetero-dimerize with partner proteins, such as NF-κB, Ras, and C/EBP. These protein–protein interactions control the regulation of pro-inflammatory target genes. A number of *in vitro* and *in vivo* studies using mouse models of inflammatory diseases demonstrate an anti-inflammatory role for GILZ. Here, authors summarize the studies that make GILZ eligible as an anti-inflammatory protein through which GCs can act. These findings permit the future development of pharmacological tools that mimic the therapeutic effects of GCs while avoiding the detrimental ones.

## Introduction

Glucocorticoid-induced leucine zipper (GILZ) is a dexamethasone-inducible gene belonging to the TSC-22 family of proteins, which are characterized by the presence of TSC box and leucine zipper domains (1). GILZ expression is rapidly and ubiquitously induced by glucocorticoids (GCs) in various types of cells, including lymphoid cells, in which it regulates activation and apoptosis. An emerging body of literature has established GILZ as an important mediator of GC anti-inflammatory effects in cell lineages and in inflammatory mouse models, by inhibiting expression of pro-inflammatory genes (2–4). This effect resides in the ability of GILZ to homo- and hetero-dimerize with known transcription factors, thereby influencing gene transcription. Some of these partner proteins are NF-κB, Raf-1, TORC2, AP-1, Ras, and C/EBPs; all of these transcription factors are pivotal players in pro-inflammatory signaling pathways. Some of them, such as NF-κB and Raf-1/Ras, are even involved in oncogenesis signaling pathways. TORC2 is specifically bound by GILZ in BCR–ABL cells, leading to apoptosis (5). GILZ has also been found to be involved in allergy, being downregulated in DCs of respiratory allergic patients or in the airways of allergen-challenged asthmatic subjects and upregulated after GC treatment (6, 7). Thus, GILZ plays a pivotal role into the inflammatory processes of various sources.

Glucocorticoids are the most used anti-inflammatory drugs by virtue of their powerful effects on the cells of the immune system. However, these drugs also exhibit detrimental side effects, which can limit their usefulness. It is commonly assumed that transactivation accounts for the onset of side effects as a consequence of the metabolic activity of GCs, and transrepression for the therapeutic effects via inhibition of pro-inflammatory proteins. Nonetheless, GILZ is one of the earliest and most GC inducible genes by transactivation, as well as other genes with anti-inflammatory properties (e.g., Iκ-B). Therefore, the transactivation process is responsible also for some anti-inflammatory effects of GCs, through the use of GILZ as a mediator (4, 8, 9).

Glucocorticoid-induced leucine zipper is currently considered an important molecular player in the multiple mechanisms of GC-influenced gene regulation of inflammation and a new focus for anti-inflammatory strategies.

## GILZ Interaction with Transcription Factors

Inhibition of the transcription factor NF-κB represents one of the mechanisms by which GCs repress gene activation without binding DNA, through an indirect pathway, in a process called transrepression (10, 11). GILZ is postulated to be a mediator of GC-induced transrepression because it can bind NF-κB and inhibit nuclear translocation of the transcription factor, in T cells and TCR-triggered thymocytes (12–14). As a consequence of this interaction, expression of some immune and inflammatory response genes is repressed. In T cells, overexpression of GILZ counteracts the NF-κB-induced up-regulation of the Fas/FasL system, thus preventing activation-induced apoptosis (1). In thymocytes, the same mechanism seems to be responsible for the inhibition of cell death, controlled by some NF-κB-regulated genes (12, 15). Very recently, in B cells, lack of GILZ has been found to cause increased NF-κB transcriptional activity with consequent contribution to the development of non-lethal B lymphocytosis (16). In bone marrow mesenchymal stem cells, GILZ inhibits COX-2 expression through inhibition of NF-κB. Furthermore, GILZ knockdown reduces GC inhibitory effect on cytokine-induced COX-2 expression, further demonstrating that GILZ can be a mediator of GC effects (17).

The interaction between GILZ and NF-κB has many other consequences. A particular, and still unexplained, interaction with NF-κB comes from studies in endothelial cells. IL-10 was found to upregulate GILZ in human umbilical vein endothelial cells (HUVEC), thus contributing to the failure to endothelium-dependent costimulation of CD4 T cells (18). In another study with the same cell type, overexpression of GILZ decreased TNF-induced transmigration of leukocytes by inhibition of NF-κB binding to DNA (19). These results would suggest that GILZ is involved in leukocyte rolling and adhesion. Interestingly, neither physical interaction between GILZ and the p65 NF-κB subunit nor inhibition of NF-κB translocation to the nucleus was observed. Thus, the exact mechanism through which GILZ inhibits NF-κB is still unclear. Similarly, another study demonstrated that endogenous expression of GILZ in HUVEC prevents vascular inflammation via inhibition of NF-κB nuclear translocation (20). Thus, NF-κB represents a key target for GILZ-mediated GC anti-inflammatory activity, and, by consequence, GILZ represents a potential target for treatment of inflammation in the endothelium.

Glucocorticoid-induced leucine zipper was also found to homo- and hetero-dimerize with AP-1 pathway components, such as c-Jun and c-Fos. GILZ can bind c-Jun and c-Fos through its N-terminal region, thereby inhibiting AP-1-driven activation. Such an interaction contributes to the regulation of AP-1 activity by GCs through a mechanism different from GR direct binding to transcription factors (21).

Although GILZ has been found to antagonize the activity of transcription factors, such as NF-κB and AP-1, it can also act as a chaperone protein. GILZ expression is induced by aldosterone in cortical collecting tubules in the kidney. Once induced, GILZ contributes to the stimulation of epithelial sodium channel (ENaC)-mediated Na+ transport via mineralocorticoids through the inhibition of the ERK cascade (22, 23). Through a second GILZ-dependent mechanism, ion transport can be controlled through physical interaction between GILZ and serum, and glucocorticoid-induced kinase 1 (SGK1). GILZ stabilizes SGK1 by recruiting it to the ER. This event inhibits SGK1 ubiquitination and, as a result, ENaC is activated (24). Therefore, GILZ represents a key regulator of transepithelial ion transport in the kidneys.

Pharmacological treatment with GCs is known to promote osteoporosis; however, osteoporosis can be induced by chronic inflammation as well (25). *GILZ*, despite being a GC-induced gene, is involved in the regulation of osteogenesis. In mesenchymal stem cells, GILZ acts as a transcriptional repressor by inhibiting C/EBP-δ-induced PPAR-γ2 expression, a key regulator of adipogenesis. GILZ binds specifically to tandem C/EBP sites in the promoter, forming complexes with C/EBP-δ and other still unknown transcription factors, thus inhibiting expression of the target genes (26, 27). Therefore, GILZ can drive the differentiation of mesenchymal stem cells toward the osteogenic pathway. These findings suggest that GILZ expression is important for osteoblast differentiation, whereas lack of GILZ can trigger the maturation of adipocytes. Overall, GILZ expression tips the balance of mesenchymal stem cell commitment in the bone marrow, playing an opposing role to GCs, which induce the differentiation toward adipocytes (28). Most likely, GILZ can temper the damage caused to bones by chronic inflammation because of its anti-inflammatory properties.

## GILZ and T Lymphocytes

**Figure 1:**
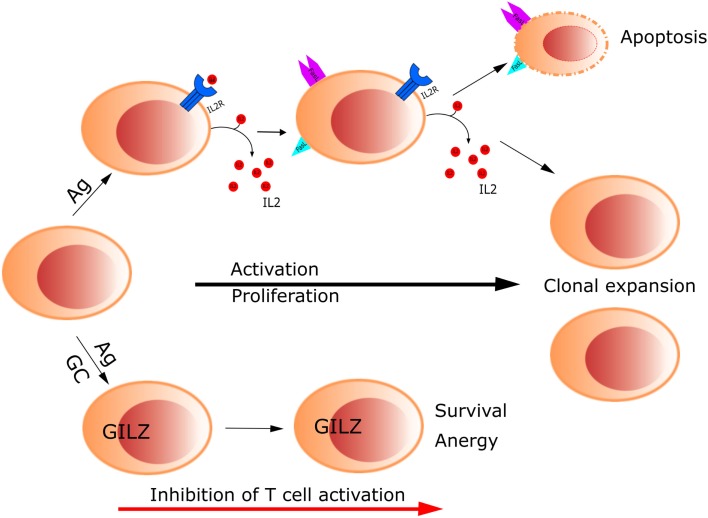
**Glucocorticoids control T cell activation through up-regulation of GILZ**. GILZ inhibits TCR-induced IL-2 and IL-2 receptor expression, which in turn inhibits Fas/FasL expression, promoting T cell survival and anergy.

Control of lymphocyte function during an immune response by means of GCs implies regulation of activation and apoptosis of T cells. GILZ was found to modulate T lymphocyte response in the murine cell line 3DO; overexpression of GILZ inhibited anti-CD3-induced apoptosis through the regulation of the Fas/FasL system (1). Thereafter, our laboratory reported that GILZ inhibits TCR-induced IL-2 and IL-2 receptor expression, which can explain the aforementioned effect on the inhibition of Fas/FasL expression and consequent T cell survival (12). These results indicate GILZ counters TCR-induced T cell activation, thus contributing to T cell anergy (Figure 1). Since then, additional studies have been published, supporting a role of GILZ as a mediator of GC action on T lymphocytes, through its function in regulating the homeostasis of these cells (3). One mechanism by which GILZ regulates T lymphocyte is through IL-2, the main survival interleukin in activated T lymphocytes. IL-2 is downregulated by GILZ, and it also inhibits *GILZ* expression through the repression of the transcription factor FoxO3, which directly acts on GILZ promoter (12, 29). Therefore, FoxO3 seems to be the mediator of GC action and IL-2 antagonism for GILZ regulation in T lymphocytes. In turn, GILZ negatively regulates FoxO factors, such as FoxO1, FoxO3, and FoxO4 (30).

Like GCs, GILZ can modulate a switch from Th1 to Th2 immune phenotypes, as demonstrated by the up-regulation and down-regulation of Th2 and Th1 cytokines, respectively. This conclusion was demonstrated in CD4^+^ cells of *GILZ* transgenic mice (TG), where an increase of IL-4 and a decrease of IFN-γ were observed. This shift in cytokine expression causes *GILZ* TG mice to be less susceptible to the induction of colitis. Much like what has been observed for the other transcription factors, GILZ can regulate the transcription of factors that control Th1- and Th2-specific cytokine expression. GILZ up-regulates GATA 3 and STAT6 to modulate the Th2 phenotype, and downregulates T-bet to modulate the Th1 phenotype (9).

GILZ plays another important role by contributing to the TGF-β-mediated generation of peripheral Treg (pTreg). GCs synergize with TGF-β signaling to increase Treg formation in upregulating Foxp3, the master regulator transcription factor of Tregs (31). *GILZ*-knock-out (KO) mice show a reduction of pTreg cells due to a diminished TGF-β signaling. As a consequence, an increased susceptibility to DNBS-induced Th1-type colitis is associated with GILZ absence. Thus, GILZ represents the means by which stress hormones encounter TGF-β signaling in the control of pTreg production (32).

Most recently, GILZ was found to regulate Th17 cells. GILZ controls the secretion of IL-17A by CD4 T cells by regulating the expression of cytokines favoring the Th17 phenotype, such as IL-1α, IL-23, and IL-6. In the mouse imiquimod model of psoriasis, in which Th17 lymphocytes play a pathogenic role, GILZ-KO mice developed excessive inflammation and exhibited enhanced pro-inflammatory cytokine expression. These results suggest that Th17 cells are another T cell subtype controlled by GILZ (33).

Glucocorticoid-induced leucine zipper is also pivotal in controlling T lymphocyte functions prior to their maturation in the thymus. Constitutive *GILZ* overexpression in *GILZ*-TG mice causes an increase in apoptosis, which is associated with an augmented activation of the caspase-8/caspase-3 pathway. As a consequence, adult *GILZ*-TG mice show a reduced number of CD4 and CD8 double positive thymocytes (34). In addition, GILZ prevents apoptosis induced by stimulation of TCR in thymocytes through the inhibition of NF-κB (15). Because GCs induce apoptosis in thymocytes and rapidly upregulate GILZ, the latter represents the mediator of GC action in the thymus.

## GILZ Function in Mouse Models of Inflammation

Because GILZ is involved in the inflammatory process, it has been studied in many of the mouse models for inflammatory diseases. Inflammatory bowel diseases (IBDs) are autoimmune/inflammatory diseases of the gastrointestinal tract that can be easily reproduced in mice. IBDs are usually treated with GCs; thus, given the relationship between GILZ and GCs, the role of GILZ has been studied in rodent models of IBDs. In TG mice overexpressing *GILZ* in CD4^+^ cells, the severity of colonic inflammation is diminished compared to the control mice. This result suggests that GILZ inhibits Th1 activation in favor of the Th2 commitment of CD4^+^ cells exerted. Furthermore, treatment of IL-10-KO mice with a recombinant TAT–GILZ protein reduces the severity of spontaneously developed colitis (8). The Th2 phenotype of *GILZ*-TG mice was shown previously in another Th2-driven disease and model of inflammation: bleomycin-induced lung injury. *GILZ-*TG mice exhibit a more severe bleomycin-induced lung injury, but they are protected under a Th1-dependent delayed-type hypersensitivity response (9). From these data, one might hypothesize that the effect of GILZ on Th2-driven differentiation overlaps with the action of GC.

Overexpression of GILZ in the TG mice has proven to be a helpful tool in the study of GILZ in another model of inflammation, i.e., the post-traumatic disease that develops after spinal cord injury (SCI). GILZ prevents the development of SCI mainly by inhibiting T cell activation and the release of pro-inflammatory cytokines, such as TNF-α and IL-1β (35). Similar effects on activated T cells were found in a mouse model of experimental autoimmune encephalomyelitis, in which a GILZ peptide (GILZ-P) interacting with NF-κB was administered on the day of disease onset. Treated mice were protected against the disease, demonstrating the powerful control GILZ can exert on T cell activity (36). Using the same inflammatory model, another study demonstrated that the induction of GILZ in dendritic cells mirrors the immunomodulation by GC in the control of autoaggressive T cell responses (37).

Anti-inflammatory actions of GILZ are evident even in mouse models of acute inflammation, such as endotoxemia. GILZ expression led to the resistance of SPRET/Ei mice to LPS-induced endotoxemia through an altered cytokine production. Furthermore, mice that received *in vivo* administration of a pES34–TAT–GILZ expression vector exhibited increased resistance to lethal effects of LPS (38). In a second example, GILZ was studied in the rodent model of LPS-induced pleurisy. Administration of recombinant TAT–GILZ protein accelerated the resolution phase of acute inflammation, reducing the magnitude of PMN infiltration by inducing apoptosis (39). Lastly, GILZ modulates acute inflammation in other cells of the innate system, the macrophages. In a mouse model for LPS tolerance, GILZ was upregulated in alveolar macrophages, whereas in endotoxin-tolerized *GILZ*-KO macrophages, cytokine induction and MAPK activation were rescued (40).

Glucocorticoid-induced leucine zipper plays a role in arthritis as well. In the CIA model of arthritis, GILZ expression mimicked the therapeutic effect of GCs, and deletion of *GILZ* increased the severity of the disease together with the expression of TNF and IL-1. Importantly, GILZ was found in the sinovium of patients with active RA, suggesting GILZ is crucial for the regulation of the local inflammatory response functioning as an endogenous inhibitor of chronic inflammation (41). Overall, these examples of GILZ involvement in inflammatory diseases clearly illustrate that GILZ acts as a mediator for the GC therapeutic effect, both in T lymphocytes and other cells of the innate immune system.

## Beyond Inflammation

**Figure 2:**
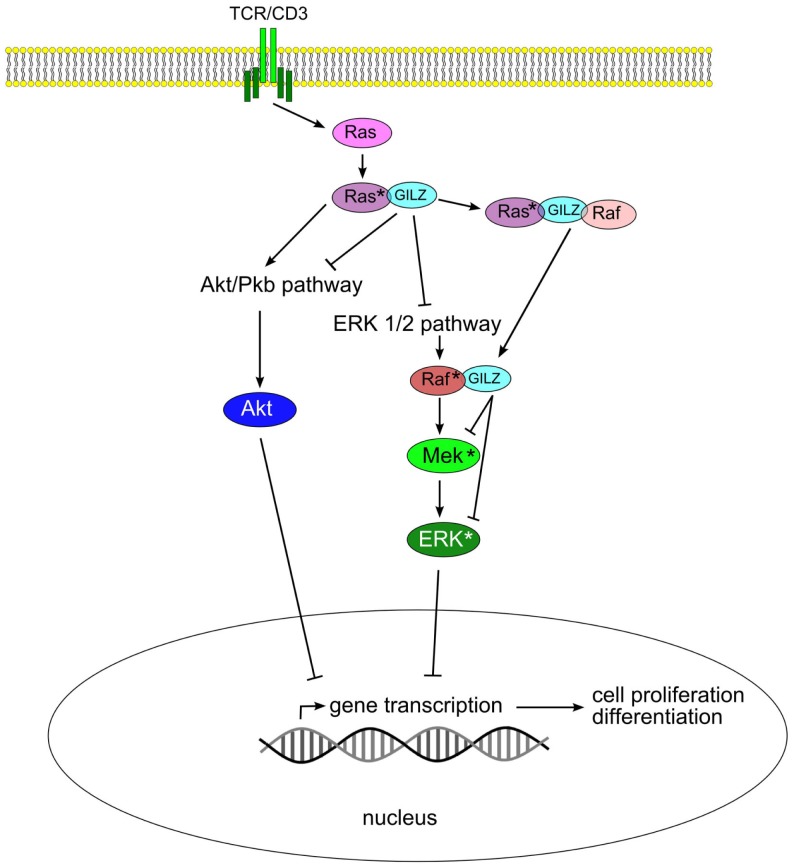
**Glucocorticoid-induced leucine zipper inhibits Akt/Pkb and ERK 1/2 pathways by binding to Ras and Raf, thus blocking cell proliferation and transformation**.

The ability of GILZ to hetero-dimerize even with “non-LZ” proteins extends its functions beyond inflammation to a number of additional pathways. GILZ can form heterodimers with Ras and Raf from the MAP-kinase/ERK-kinase pathway, as well as ternary complex with both binding partners, thereby controlling growth of the cell (42). Signaling through the Ras family of small GTPases is pivotal in the transmission of mitogenic stimuli to the cell cycle machinery. The fact that GILZ binds predominantly to activated Ras, but not to Raf, suggests a fine tuning of the regulation of this pathway. Interestingly, GILZ binds to Raf when Ras in not activated. Thus, in the case of activated Ras, both Akt/Pkb serine (threonine kinase) and ERK pathways are inhibited by GILZ. When Ras is not activated, GILZ can influence only the ERK pathway via interaction with Raf (Figure 2).

## Conclusion

In recent years, GILZ has emerged as a pivotal mediator of GC action in the inflammatory process, as demonstrated by many *in vitro* and *in vivo* studies. This GILZ function opens the possibility to develop new or repurposed pharmaceutical tools for the treatment of inflammation-based diseases. These new therapies could achieve obtaining the same effect as GC treatment, but circumvent the undesirable side effects caused by GCs.

## Conflict of Interest Statement

The authors declare that the research was conducted in the absence of any commercial or financial relationships that could be construed as a potential conflict of interest.
